# Supplementary material for: gbpA and chiA genes are not uniformly distributed amongst diverse Vibrio cholerae
Source: Microb Genom. 2021 Jun 8;7(6):000594. doi: 10.1099/mgen.0.000594 (PMC8461464; doi:10.1099/mgen.0.000594)
Supplement: Supplementary material 1 [file mgen-7-0594-s001.pdf]

Supplementary material for

gbpA and *chiA* genes are not uniformly distributed amongst diverse *Vibrio cholerae*

Thea G. Fennell, Grace A. Blackwell, Nicholas R. Thomson & Matthew J. Dorman

These supplementary materials include:

Supplementary Table 1 (separate .xls file)

Supplementary Tables 2-3

Supplementary Figures 1-7

Supplementary References

Additional materials that support this study are available in Figshare:

https://dx.doi.org/10.6084/m9.figshare.13169189

Supplementary Table 2. Summary of seven previously-published putative and validated chitinase genes from N16961 [1] annotated in the *V. cholerae* pangenome. Order corresponds to that of Figure 2. Gene cluster IDs correspond to the gene presence/absence matrix available in the Figshare repository supporting this study. The dataset contains a total of 198 genomes, of which three are not *V. cholerae*.

**Gene cluster ID**	**N16961 locus ID**	**# isolates containing gene cluster (198 total)**	**Details**
gbpA	*VC_A0811*	189	Encodes GbpA
endo I_1	*VC_1073*	196	Putative chitinase
group_316	*VC_A0700*	195	Encodes chitodextrinase
endo I_3	*VC_0769*	198	Putative chitinase
chiD	*VC_1952*	131	Encodes ChiA-1
chiA	*VC_A0027*	194	Encodes ChiA-2
chiA_2	*VC_A0140*	194	Putative chitinase

Supplementary Table 3. All genes annotated in the *V. cholerae* pangenome as ‘chitinase’ or ‘putative chitinase’. Gene cluster IDs correspond to the gene presence/absence matrix available in the Figshare repository supporting this study. The dataset contains a total of 198 genomes, of which three are not *V. cholerae*.

**Gene cluster ID**	**Annotation**	**# isolates containing gene cluster (198 total)**	**N16961 locus ID**
endo I_3	putative chitinase	198	*VC_0769*
endo I_1	putative chitinase	196	*VC_1073*
chiA	chitinase	194	*VC_A0027* (*chiA-2*)
chiD	chitinase	131	*VC_1952* (*chiA-1*)
group_1311	chitinase	87	n/a (*chiA-3*)
endo I_2	chitinase	45	n/a (no chitinase domain)
group_14144	chitinase	1	n/a
VCJ_003148	chitinase	1	n/a
VCJ_001093	chitinase	1	n/a
VOA_002115	chitinase	1	n/a
VOA_001161	chitinase	1	n/a

Supplementary Figures

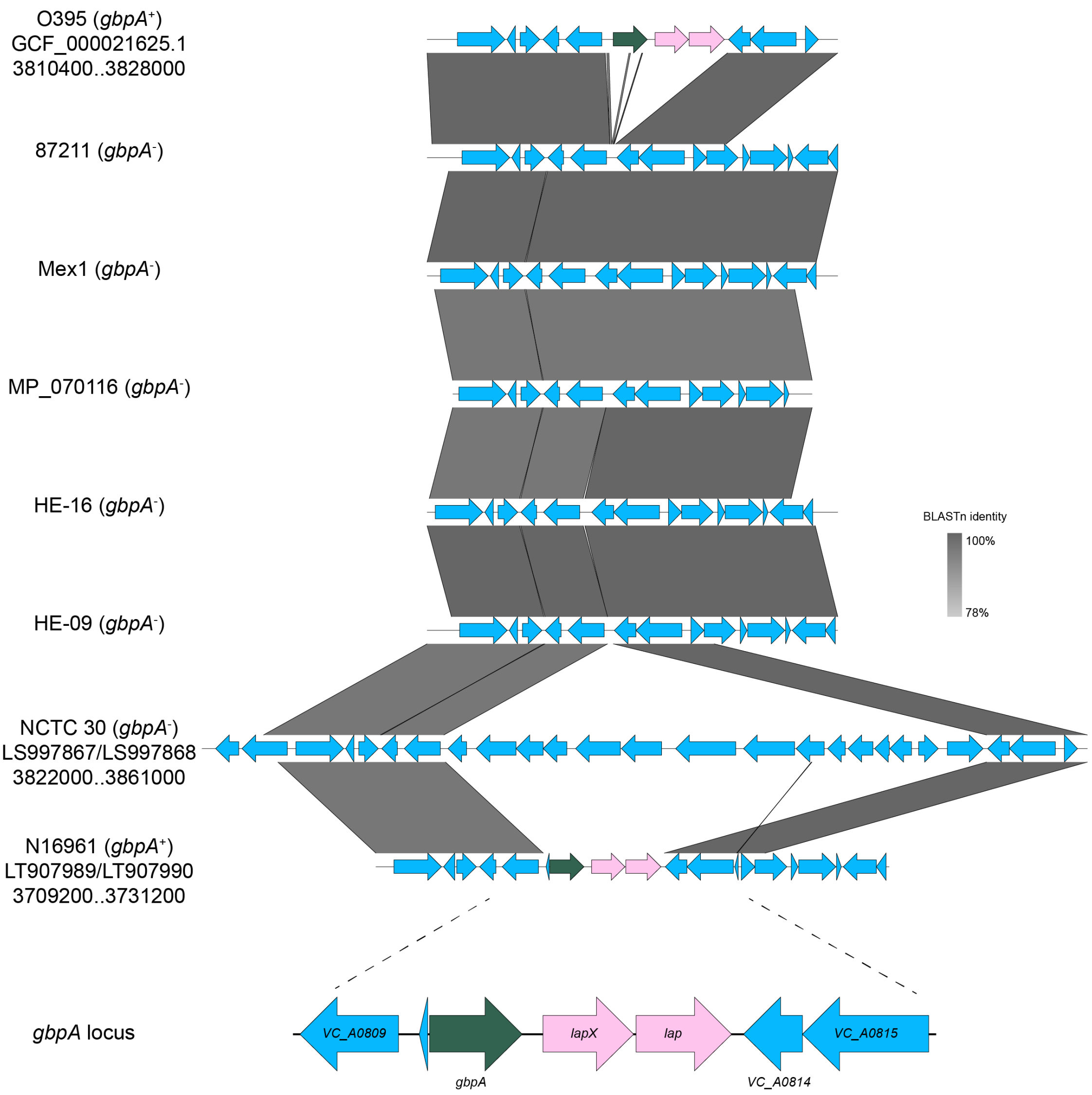

Supplementary Figure 1. Confirming the absence of *gbpA* and adjacent genes from assemblies. Heterogeneity at the genomic locus encompassing *VC_A0811* was observed in isolates lacking *gbpA*. All assemblies lacked *VC_A0811*-*VC_A0813*, and these genes were replaced with sequence containing at least 15 genes in NCTC 30. Read mapping data confirming the absence of *VC_A0811*-*VC_A0813* from NCTC 30 are presented in Supplementary Figure 2. Accession numbers and assembly co-ordinates are reported for reference and closed genome sequences. Figure generated using Easyfig [4] and BLASTn comparisons [5]. *VC_A0811*-*VC_A0813* are highlighted in N16961 and O395 (both *gbpA*$^{+}$) for ease of illustration.

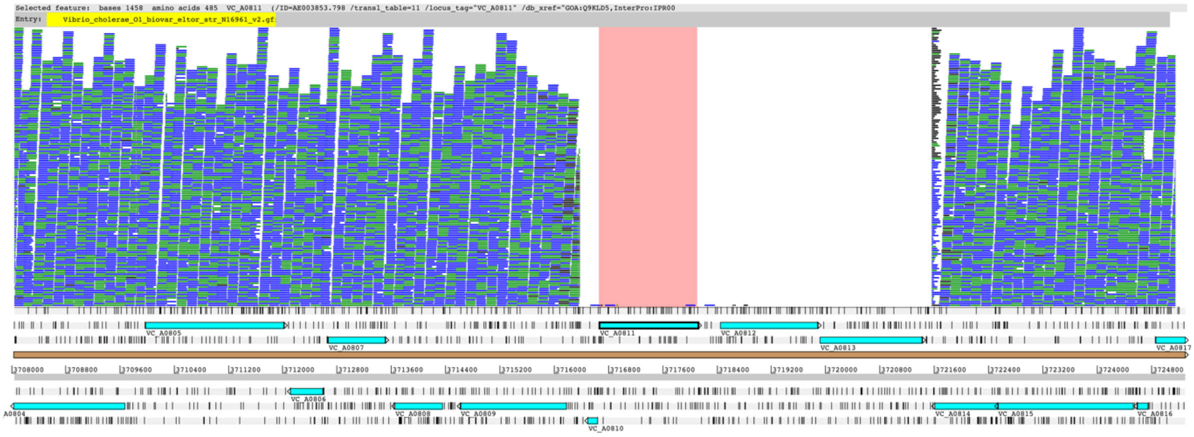

Supplementary Figure 2. Confirming the absence of *VC_A0811-VC_A0813* from NCTC 30 by mapping. Illumina short-reads from NCTC 30 were mapped to the N16961 reference sequence and visualized using Artemis and BamView [2, 3]; *gbpA* is highlighted. The drop in coverage over these genes indicates that these are absent from the sequenced NCTC 30 genome.

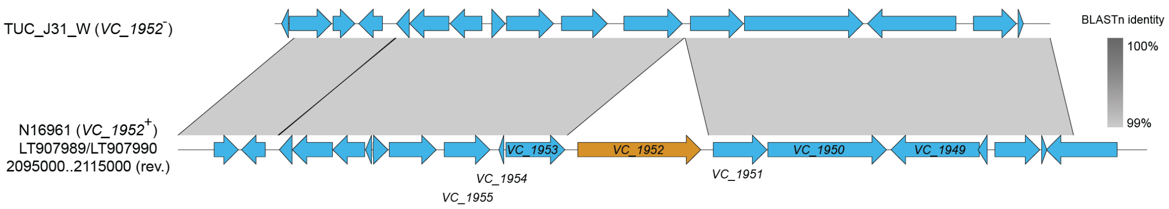

Supplementary Figure 3. Genes adjacent to the *VC_1952* locus are intact in genomes lacking *chiA-1*. An example is presented in which the genes flanking *VC_1952* remain intact in the absence of *VC_1952* itself, contrasting with the observation made at the *gbpA* locus (Supplementary Figure 1). A larger number of diverse genomes are similarly analyzed in Supplementary Figure 4. Figure generated using Easyfig [4] and BLASTn comparisons [5].

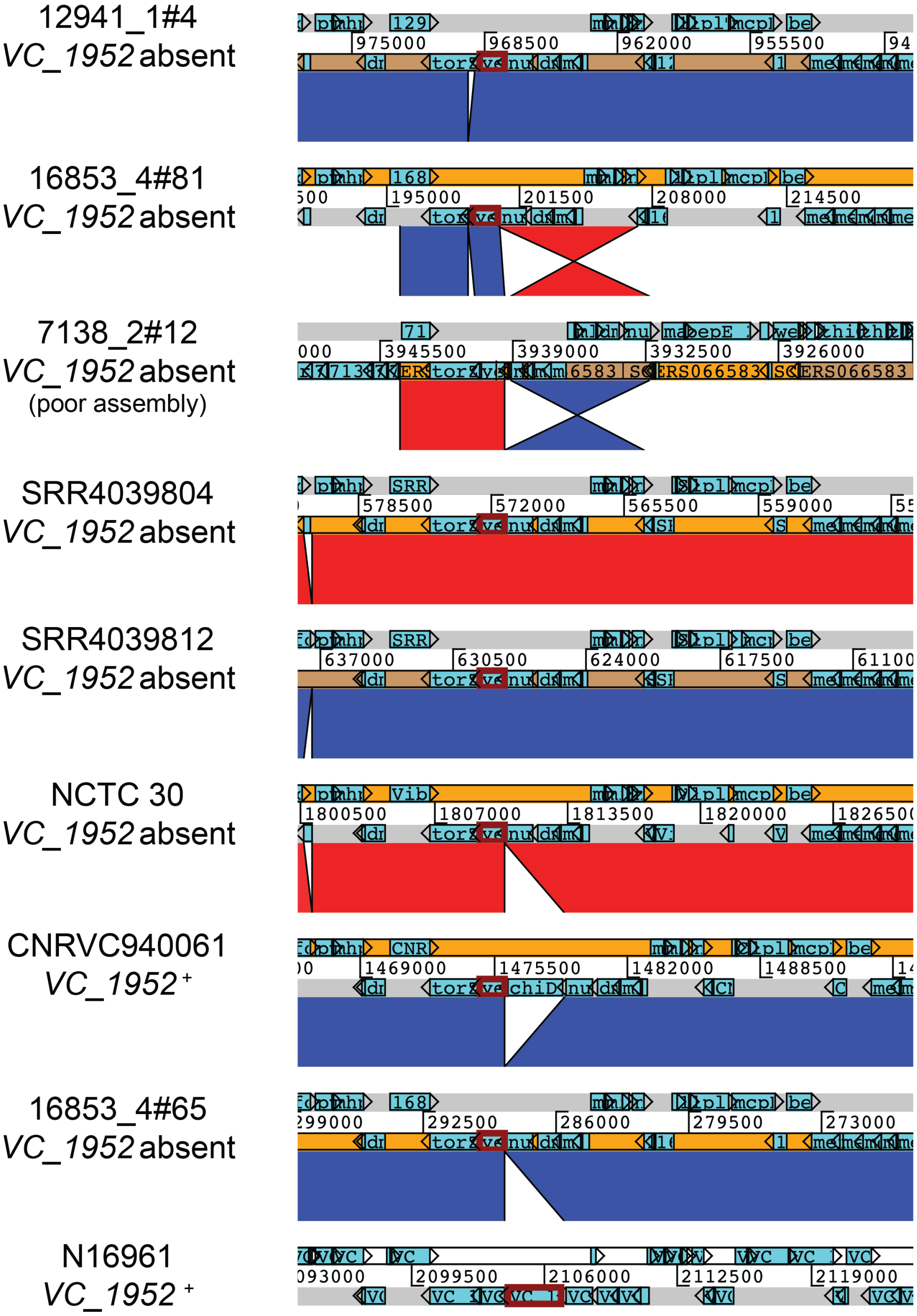

Supplementary Figure 4. Loss of *VC_1952* in multiple diverse *V. cholerae*. ACT comparisons of assemblies for a set of seven *V. cholerae* lacking *chiA-1* and two which harbour *chiA-1*, aligned using BLASTn [5]. In all cases, the absence of *chiA-1* (*VC_1952*) does not interfere with the genes surrounding this locus. The *chiA-1* gene has been highlighted in the N16961 reference sequence (bottom of figure), and syntenic contigs have been aligned.

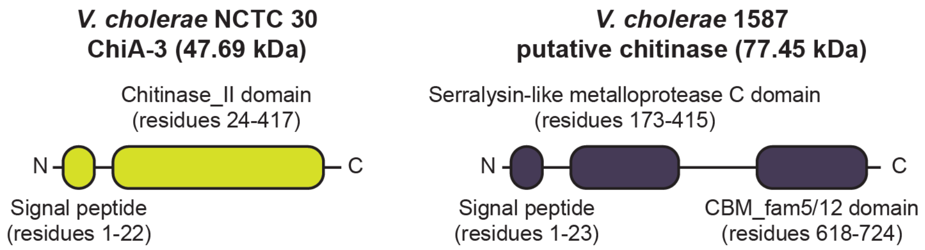

Supplementary Figure 5. Two putative chitinases are of different sizes and contain different predicted functional domains. Domain predictions were derived from InterProScan [6] using translated protein sequences obtained from NCTC 30 (gene cluster 'group_1311'; Figure 2, Supplementary Table 3) and *V. cholerae* 1587 (gene cluster 'endo_I2') genome sequences. Images are not to scale.

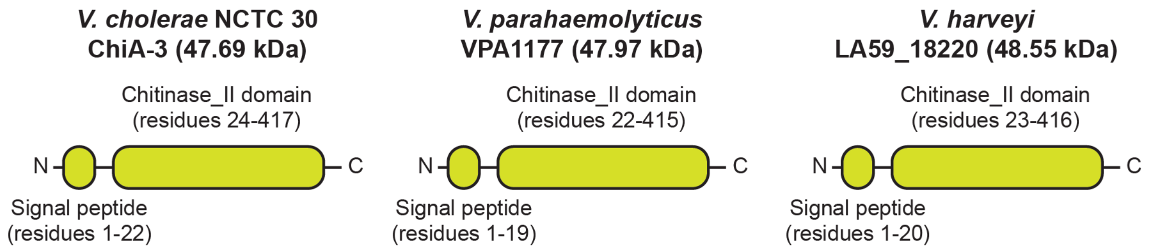

Supplementary Figure 6. ChiA-3 orthologues from *V. parahaemolyticus* and *V. harveyi* are predicted to have similar domain structures and molecular weights to ChiA-3 from *V. cholerae*. Domain predictions were derived from InterProScan [6] using protein sequences obtained from the annotated genomes presented in Figure 3b [7, 8]. Images are not to scale.

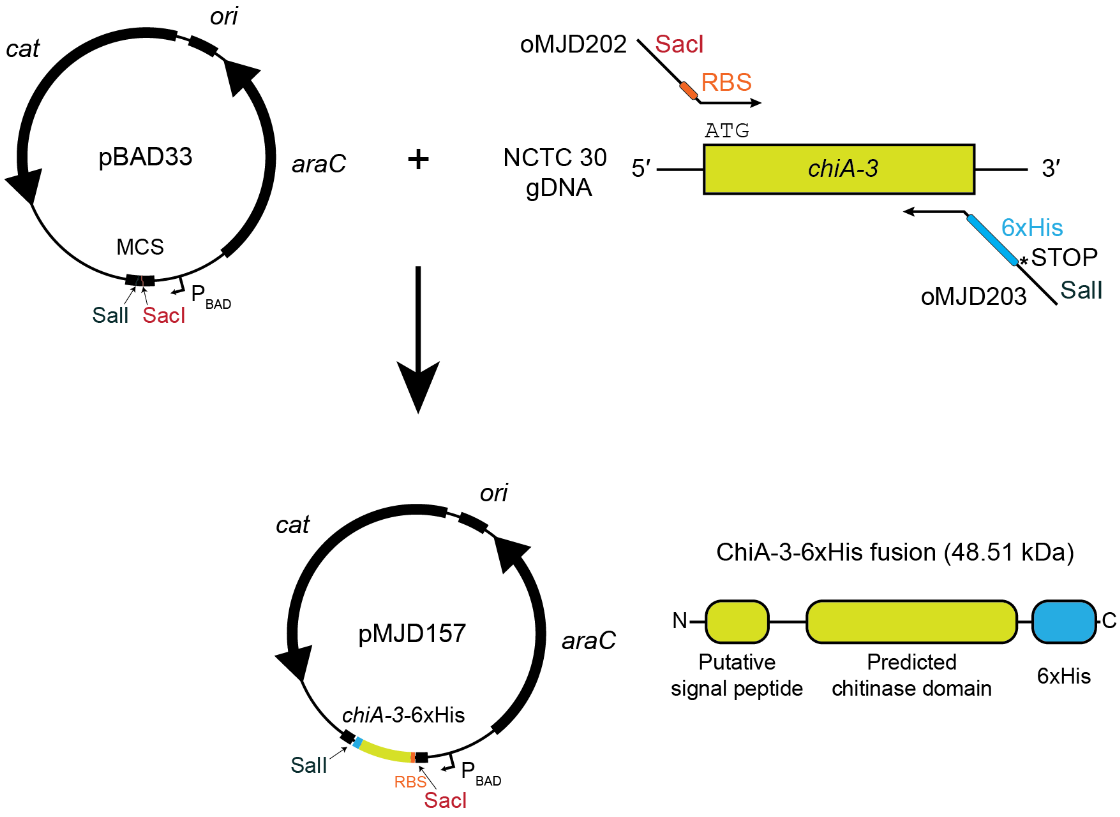

Supplementary Figure 7. Strategy for construction of pMJD157. Schematic of cloning strategy used to amplify and insert *chiA-3* directionally from NCTC 30 gDNA into the pBAD33 multiple cloning site (MCS), under the arabinose-inducible P_{BAD} promoter, and to incorporate a C-terminal 6xHis tag into ChiA-3 as a translational fusion. A linker sequence was not incorporated between the C-terminus of ChiA-3 and the 6xHis tag. Figures are not to scale.

Supplementary References

1. **Hayes CA, Dalia TN, Dalia AB**. Systematic genetic dissection of chitin degradation and uptake in *Vibrio cholerae*. *Environ Microbiol* 2017;19:4154–4163.

2. **Carver T, Böhme U, Otto TD, Parkhill J, Berriman M**. BamView: Viewing mapped read alignment data in the context of the reference sequence. *Bioinformatics* 2010;26:676–677.

3. **Rutherford K, Parkhill J, Crook J, Horsnell T, Rice P, *et al.*** Artemis: Sequence visualization and annotation. *Bioinformatics* 2000;16:944–945.

4. **Sullivan MJ, Petty NK, Beatson SA**. Easyfig: A genome comparison visualizer. *Bioinformatics* 2011;27:1009–1010.

5. **Altschul SF, Gish W, Miller W, Myers EW, Lipman DJ**. Basic local alignment search tool. *J Mol Biol* 1990;215:403–410.

6. **Jones P, Binns D, Chang H-Y, Fraser M, Li W, *et al.*** InterProScan 5: Genome-scale protein function classification. *Bioinformatics* 2014;30:1236–1240.

7. **Makino K, Oshima K, Kurokawa K, Yokoyama K, Uda T, *et al.*** Genome sequence of *Vibrio parahaemolyticus*: a pathogenic mechanism distinct from that of *V cholerae*. *Lancet* 2003;361:743–749.

8. **Wang Z, Hervey WJ, Kim S, Lin B, Vora GJ**. Complete genome sequence of the bioluminescent marine bacterium *Vibrio harveyi* ATCC 33843 (392 [MAV]). *Genome Announc* 2015;3:e01493-14. DOI: 10.1128/genomeA.01493-14.
